# Multiplication and division of the orbital angular momentum of light with diffractive transformation optics

**DOI:** 10.1038/s41377-019-0222-2

**Published:** 2019-12-05

**Authors:** Gianluca Ruffato, Michele Massari, Filippo Romanato

**Affiliations:** 1 Department of Physics and Astronomy ‘G. Galilei’, University of Padova, via Marzolo 8, 35131 Padova, Italy; 2 LaNN, Laboratory for Nanofabrication of Nanodevices, EcamRicert, Corso StatiUniti 4, 35127 Padova, Italy; 3 CNR-INFM TASC IOM National Laboratory, S.S. 14 Km 163.5, 34012 Trieste, Italy

**Keywords:** Integrated optics, Transformation optics, Transformation optics

## Abstract

We present a method to efficiently multiply or divide the orbital angular momentum (OAM) of light beams using a sequence of two optical elements. The key element is represented by an optical transformation mapping the azimuthal phase gradient of the input OAM beam onto a circular sector. By combining multiple circular-sector transformations into a single optical element, it is possible to multiply the value of the input OAM state by splitting and mapping the phase onto complementary circular sectors. Conversely, by combining multiple inverse transformations, the division of the initial OAM value is achievable by mapping distinct complementary circular sectors of the input beam into an equal number of circular phase gradients. Optical elements have been fabricated in the form of phase-only diffractive optics with high-resolution electron-beam lithography. Optical tests confirm the capability of the multiplier optics to perform integer multiplication of the input OAM, whereas the designed dividers are demonstrated to correctly split up the input beam into a complementary set of OAM beams. These elements can find applications for the multiplicative generation of higher-order OAM modes, optical information processing based on OAM beam transmission, and optical routing/switching in telecom.

## Twisted light: multiplication and division of orbital angular momentum

A compact optical system for manipulating the orbital angular momentum (OAM) of light has been developed by researchers in Italy. The OAM carried by certain twisted light beams has applications including manipulating particles and transmitting data, but there is a urgent need for devices that can control and change the OAM state of light. In particular, so far the multiplication or division of the OAM state has required bulky systems with many optical components that are not suitable for miniaturization. Now, Gianluca Ruffato at the University of Padova and coworkers have used electron-beam lithography to fabricate compact diffractive optics components that can efficiently multiply or divide the OAM state of light. The system should enable the generation of higher-order OAM modes for optical information processing and the optical routing of signals in telecommunications.

## Introduction

Since the seminal paper of Allen and coworkers^1^, light beams carrying orbital angular momentum (OAM) gave rise to a flourishing field of research, leading to a rich multiplicity of studies and applications:^2^ particle trapping, tweezing, and manipulation^3^, high-resolution microscopy^4,5^, astronomical coronagraphy^6^, mode-division multiplexing^7^, and security^8,9^. Concurrently, several optical methods and devices have been described and engineered to tailor and control the OAM content of light beams, such as spiral phase plates^10,11^, computer-generated holograms^12,13^, *q*-plates^14^, transformation optics^15,16^, and metasurfaces^17^. Basically, all those methods rely on transferring an azimuthal phase gradient *exp*(*iℓϑ*) to the input beam, with *ϑ* being the polar angle on a plane transverse to the propagation direction and *ℓ* being the amount of OAM per photon in units of ℏ. In microscopy, a beam carrying OAM was used to suppress fluorescence around the dark central zone, providing sub-wavelength lateral confinement and consequent super-resolution, as shown by the pioneering paper of Hell et al.^18^. In the telecom field, the potentially unbounded state space provided by this even-unexploited degree of freedom offers a promising solution to increase the information capacity of optical networks^19^, both for free space^20^ and optical fiber^21^ propagation. At present, it is urgent to further develop novel devices that can dynamically reconfigure and switch between distinct OAM modes^22,23^ to fully exploit the extra degree of freedom provided by the OAM for both classical and quantum communications. The abovementioned conventional methods are useful for implementing only shift operations on the OAM mode, i.e., addition or subtraction. On the other hand, it would be extremely beneficial to be able to either multiply or divide the OAM state for some applications, such as the multiplicative generation of high-order modes, optical switching, and routing^24,25^, and optical OAM-based information processing^26,27^.

To date, OAM multiplication and division have been implemented by means of bulky and sophisticated solutions^28–30^, which are barely suitable for integration and miniaturization owing to the presence of many optical elements. Multiplication has been performed^28,29^ by mapping the input azimuthal phase gradient into a linear phase gradient with a first *log-pol* sorter^31,32^, then creating multiple copies with an optical fan-out^33^, and finally wrapping the extended phase gradient into a doughnut shape with a second *log-pol* sorter, working in reverse, for a total of at least six optical elements, plus lenses in between for the Fourier transform and beam resizing. Division has been implemented in a similar manner^29,30^ by substituting the multiple-copy stage with a mask for the selection of only a fraction of the linear phase gradient in the input, at the expense, therefore, of a non-negligible amount of the input energy. In the quest for miniaturization, the implementation of the *log-pol* transformation stages in the form of diffractive optics^34^ and the integration of the copying/fractioning operation in the *log-pol* optics^35^ could improve the compactness of the system. However, the number of optical operations, i.e., *log-pol* mapping, phase-resizing, and inverse transformation, would remain the same.

Here, we present a completely different method, which basically preserves the axial symmetry and avoids the limitations of the *log-pol* coordinate-change approach. The key element is represented by an optical transformation that maps the azimuthal phase gradient of the input OAM beams into a circular sector. By combining multiple circular-sector transformation onto a single optical element, it is possible to multiply the input OAM by mapping its phase onto complementary circular sectors. Conversely, by combining multiple inverse transformations, it is possible to map different complementary sectors of the input beam into an equal number of circular phase gradients, thus achieving a division of the initial OAM. These operations can be realized by a sequence of only two elements, performing the optical transformation of the beam and the required phase correction. This approach allows for the multiplication and division of the OAM in a compact manner, remarkably reducing the number of optical operations and the total number of optical elements and therefore providing a final significant increase in the optical efficiency. The designed optical elements have been fabricated in the form of phase-only diffractive optics with high-resolution electron-beam lithography and optically characterized to demonstrate the expected capability to either multiply or divide the OAM of the input beam. In addition, a compact optical architecture has been presented to arrange the two optical elements required for optical transformation and phase correction onto the same substrate, further improving alignment and miniaturization.

## Results

### Theory and simulation

In the following paragraphs, the theory underlying multiplication and division with transformation optics is presented and described. At first, the key element of these operations, i.e., the circular-sector transformation, is introduced and developed in the paraxial approximation. Therefore, the possibility of combining many circular-sector transformations to either multiply or divide the OAM content of an input beam is shown, and the phase patterns of the corresponding optical elements are calculated.

#### Circular-sector transformation

**Fig. 1:**
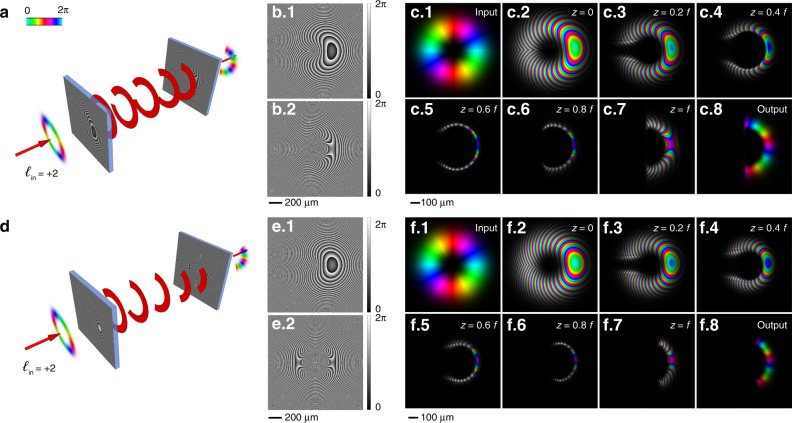
Circular-sector transformation of OAM beams. Schematics of OAM beam transformation with circular transformation optics for twofold (*n* = 2) **a** and threefold (*n* = 3) **d** circular-sector transformation. The first phase pattern (**b**.1, **e**.1) performs an *n*-fold circular-sector transformation, mapping the input intensity distribution onto a 2π/*n* arc. The second phase pattern (**b**.2, **e**.2) performs the required phase correction, retaining the compressed azimuthal phase distribution. Design parameters: *a* = 300 μm, *b* = 250 μm, and *f* = 20 mm. Numerical simulations of the propagation of an input Laguerre-Gaussian beam carrying *ℓ* = 2 (**c**.1, **f**.1), after illuminating the first element (**c**.2, **f**.2), at different *z* positions (*z* = 0.2 *f* (**c**.3, **f**.3), *z* = 0.4 *f* (**c**.4, **f**.4), *z* = 0.6 *f* (**c**.5, **f**.5), *z* = 0.8 *f* (**c**.6, **f**.6), up to the second optical element (**c**.7, **f**.7), and the output phase-corrected beam (**c**.8, **f**.8). Colors and brightness refer to the phase and intensity, respectively.

The optical layout of the system consists of a cascade of two optical elements: the former performs a conformal optical transformation, whereas the latter corrects the phase distortion owing to the different paths traveled by the distinct points of the beam and restores the desired phase profile. Conversely, owing to the invariance of the light path for time reversal, the second optical element performs the inverse optical transformation. The key element of OAM multiplication and division is represented by an optical transformation performing a conformal mapping of the whole circle onto a circular sector (Fig. 1a, d)^36^. By indicating with (*r, ϑ*) the polar coordinates on the input plane and with (*ρ, φ*) the polar reference frame on the second plane, this transformation operates a rescaling of the azimuthal coordinate:

1 $$\varphi = \frac{\vartheta }{n}$$

After imposing the condition of conformity, the new radial coordinate is given by:

2 $$\rho = a\left( {\frac{r}{b}} \right)^{ - \frac{1}{n}}$$

with *a* and *b* being scaling parameters. By applying the previous transformation, an azimuthal phase gradient is mapped conformally onto a circular sector with amplitude 2π/*n*. To calculate the phase pattern of an optical element performing this transformation in the paraxial regime, we apply the stationary phase approximation^37^ to the Fresnel integral. The field *U*(*ρ*, *φ*) after a propagation length *f* along the coordinate *z* for an input plane-wave illuminating a phase-only optical element with phase function Ω_*S*,*n*_, located at *z* = 0, is given by:

3 $$U(\rho ,\varphi ) = \frac{{e^{ik\frac{{\rho ^2}}{{2f}}}}}{{i\lambda f}}{\int\!\!\!\!\!\int} {e^{i\Omega _{S,n}\left( {r,\vartheta } \right)}e^{ik\frac{{r^2}}{{2f}}}e^{ - ik\frac{{r\rho }}{f}\cos \left( {\vartheta - \varphi } \right)}rdrd\vartheta }$$

According to the stationary phase approximation^37^, the integral solution reduces to find the saddle points of the phase function in Eq. (3):

4 $$\Phi \left( {r,\vartheta } \right) = \Omega _{S,n}\left( {r,\vartheta } \right) + k\frac{{r^2}}{{2f}} - k\frac{{r\rho }}{f}\cos \left( {\vartheta - \varphi } \right)$$

The stationary condition $\nabla \Phi = 0$ leads to a system of partial derivatives of Ω_*S,n*_ that is unknown. Substituting the transformation relations, i.e., Eqs. (1) and (2), into the partial derivatives of Eq. (4) and imposing the stationary condition, we obtain:

5 $$\left\{ {\begin{array}{*{20}{c}} {\frac{{\partial \Omega _{S,n}}}{{\partial r}} + k\frac{r}{f} - k\frac{a}{f}\left( {\frac{r}{b}} \right)^{ - \frac{1}{n}}\cos \left( {\vartheta - \frac{\vartheta }{n}} \right) = 0} \\ {\frac{{\partial \Omega _{S,n}}}{{\partial \vartheta }} + k\frac{{ar}}{f}\left( {\frac{r}{b}} \right)^{ - \frac{1}{n}}\sin \left( {\vartheta - \frac{\vartheta }{n}} \right) = 0} \end{array}} \right.$$

After integrating, we obtain the analytical expression for the phase function in the paraxial regime:

6 $$\Omega _{S,n}(r,\vartheta ) = \frac{{2\pi ab}}{{\lambda f}}\left( {\frac{r}{b}} \right)^{1 - \frac{1}{n}} \cdot \frac{{\cos \left[ {\vartheta \left( {1 - \frac{1}{n}} \right)} \right]}}{{1 - \frac{1}{n}}} - k\frac{{r^2}}{{2f}}$$

The corresponding phase corrector can be calculated by applying the same method to the inverse transformation. We obtain:

7 $$\Omega _{PC,n}(\rho ,\varphi ) = \frac{{2\pi ab}}{{\lambda f}}\left( {\frac{\rho }{a}} \right)^{1 - n} \cdot \frac{{\cos \left[ {\varphi \left( {1 - n} \right)} \right]}}{{1 - n}} - k\frac{{\rho ^2}}{{2f}}$$

which is basically the expression in Eq. (6) after the substitutions *b*↔*a*, *n*→1/*n*, and (*r*, *ϑ*)→(*ρ*, *φ*). Further details regarding the calculations of Eq. (6) and Eq. (7) are provided in Supplementary Material S1.

In Fig. 1a, d, schematics of the OAM beam evolution in the case of circular-sector transformations by a factor of *n* = 2 and *n* = 3 are depicted, respectively. The phase gradient of the input OAM beam is mapped over half and one-third of the entire circle, respectively. Numerical simulations are also reported (Fig. 1c, f), showing the evolution of the beam after illuminating the first optical element and during propagation in the range of the focal length, where the second element is placed for phase correction.

#### The OAM multiplier

OAM multiplication by a factor *n* basically consists of splitting the input beam into *n* copies and mapping each one over a set of *n* complementary circular sectors. Therefore, the phase pattern Ω_*M,n*_ of the multiplier is described by the superposition of *n* circular-sector transformations {Ω_*S,n*_^(*j*)^}, *j* = 1, …, *n*, mapping the input beam over the corresponding circular sectors with amplitude 2π/*n* and centered on {(*j* – 1)2π/*n*}:

8 $$\Omega _{M,n}(r,\vartheta ) = \arg \left\{ {\mathop {\sum}\limits_{j = 1}^n {e^{i\Omega _{S,n}^{(j)}}} } \right\}$$

where:

9 $$\Omega _{S,n}^{\left( j \right)}(r,\vartheta ) = \frac{{2\pi ab}}{{\lambda f}}\left( {\frac{r}{b}} \right)^{1 - \frac{1}{n}} \cdot \frac{{\cos \left[ {\vartheta \left( {1 - \frac{1}{n}} \right) + \left( {j - 1} \right)\frac{{2\pi }}{n}} \right]}}{{1 - \frac{1}{n}}} - k\frac{{r^2}}{{2f}}$$

**Fig. 2:**
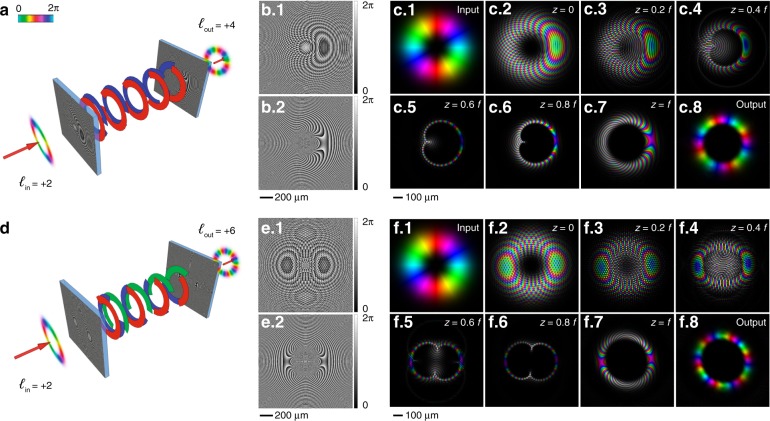
Multiplication of OAM beams. Schematics of OAM beam optical multipliers for twofold (*n* = 2) **a** and threefold (*n* = 3) **d** multiplication with transformation optics. The first phase pattern (**b**.1, **e**.1) performs an *n*-fold multiplication, splitting, and mapping the input azimuthal phase gradient over *n* complementary arcs. The second phase pattern (**b**.2, **e**.2) performs phase correction, retaining the azimuthal phase distribution. Design parameters: *a* = 300 μm, *b* = 250 μm, and *f* = 20 mm. Numerical simulations of the propagation of an input Laguerre-Gaussian beam carrying *ℓ* = 2 (**c**.1, **f**.1), after illuminating the first element (**c**.2, **f**.2), at different *z* positions (*z* = 0.2*f* (**c**.3, **f**.3), *z* = 0.4*f* (**c**.4, **f**.4), *z* = 0.6*f* (**c**.5, **f**.5), *z* = 0.8*f* (**c**.6, **f**.6), up to the second optical element (**c**.7, **f**.7), and the output phase-corrected beam (**c**.8, **f**.8). Colors and brightness refer to the phase and intensity, respectively.

As demonstrated in Supplementary Material S2, the phase corrector of an *n*-fold circular transformation does not change if a shift of *j*2π/*n*, where *j* is an integer value, is performed on the azimuthal coordinate after rescaling. Therefore, all the circular-sector transformations {Ω_*S,n*_^(*j*)^} described by Eq. (9) share the same pattern for the phase corrector, which is given again by Eq. (7). In fact, the phase-corrector phase patterns in Fig. 2b.2 and Fig. 2e.2 of the twofold and threefold multipliers, respectively, are the same as those shown in Fig. 1b.2 and Fig. 1e.2 for the constituent twofold and threefold circular-sector transformations, respectively.

In Fig. 2a, d, schematics of the beam evolution in the case of multiplication by a factor of *n* = 2 and *n* = 3 are depicted, respectively. In Fig. 2c, f, the corresponding numerical simulations are reported, providing the evolution of the beam in the range of the focal length, where the second element is placed for the phase correction. As expected, the input beam is split into *n* equal contributions that are mapped onto complementary arcs, thus performing an *n*-fold multiplication of the input OAM. A conversion efficiency close to the unit can be achieved, which gradually decreases with increasing input OAM value (Supplementary Material S4) owing to a slight distortion of the output intensity ring distribution. In Fig. 2f.8, a slight deformation of the output beam can be observed, the shape of which is not perfectly axially symmetric. As discussed and demonstrated in detail in Supplementary Material S3, this distortion is caused by the twisted wavefront of the input OAM beam. In fact, the phase patterns in Eqs. (6) and (7) are calculated in the stationary phase approximation, assuming a uniform phase front in the input. On the other hand, OAM beams are endowed with a peculiar azimuthal phase, which affects the final intensity distribution of the transformed beam. However, as shown in the Supplementary Material, this effect can be mitigated and the conversion efficiency can be optimized by properly designing the transformation optics in terms of the focal length *f* and design parameters *a* and *b*.

#### The OAM divider

**Fig. 3:**
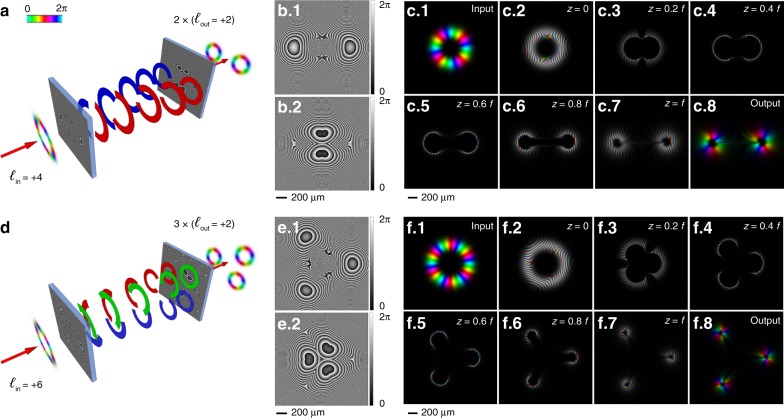
Division of OAM beams. Schematics of OAM-beam optical dividers for twofold (*n* = 2) **a** and threefold (*n* = 3) **d** division with transformation optics. The first phase pattern (**b**.1, **e**.1) performs an *n*-fold division, splitting the input azimuthal gradient into *n* complementary arcs that are wrapped and mapped over distinct whole circles at desired positions. The second phase pattern (**b**.2, **e**.2) performs phase correction, retaining the azimuthal phase distribution for each output beam. Design parameters: *a* = 300 μm, *b* = 300 μm, *f* = 20 mm, and spatial frequency *β*^(*j*)^ = 0.3 μm^−1^, *j* = 1, .., 3. Numerical simulations of the propagation of an input Laguerre-Gaussian beam carrying *ℓ* = 4 (**c**.1) or *ℓ* = 6 (**f**.1), after illuminating the first element (**c**.2, **f**.2), at different *z* positions (*z* = 0.2 *f* (**c**.3, **f**.3), *z* = 0.4*f* (**c**.4, **f**.4), *z* = 0.6 *f* (**c**.5, **f**.5), *z* = 0.8 *f* (**c**.6, **f**.6), up to the second optical element (**c**.7, **f**.7), and the output phase-corrected beams (**c**.8, **f**.8). Colors and brightness refer to the phase and intensity, respectively.

It could be interesting to divide the input beam into a bunch of *n* beams with OAMs equal to 1/*n* the OAM of the input beam, as depicted in Fig. 3a, d, for the case of twofold (*n* = 2) and threefold (*n* = 3) division, respectively. The OAM division operation can be understood considering OAM multiplication, e.g., Fig. 2d, in reverse. In Fig. 2d, the output threefold OAM beam is the combination of three complementary circular sectors of size 2π/3 that arise from the same number of combined threefold circular-sector transformations of the input beam (threefold multiplier). Thus, the input OAM beam carrying *ℓ* = 2 is split into three copies that are un-wrapped and projected onto three complementary arcs to achieve the final *ℓ* = 6 OAM beam. Considering the optical path in reverse, the three complementary circular sectors with size 2π/3 forming the OAM beam with *ℓ* = 6 are wrapped by a circular-sector transformation with *n* = 1/3 onto three OAM beams carrying the OAM value *ℓ*/3 = 2. By applying different tilt angles to the three arcs, it is possible to obtain three non-overlapping OAM beams as the output, as shown in Fig. 3d. Generalizing this approach to a division into *n* OAM beams, this is possible by defining the phase pattern of the OAM divider in the following way:

10 $$\Omega _{D,n}(r,\vartheta ) = \arg \left\{ {\mathop {\sum}\limits_{j = 1}^n {e^{i\Omega _{S,1/n,j}\left( {r,\vartheta } \right)}e^{ir \cdot \beta ^{(j)}\cos \left( {\vartheta - \vartheta ^{(j)}} \right)}} \Phi _n^{(j)}\left( \vartheta \right)} \right\}$$

where Ω_*S,1/n,j*_ is the phase pattern of a 1/*n*-fold circular-sector transformation centered on (*j* − 1)2π/*n*, *j* = 1, …, *n*:

11 $$\Omega _{S,1/n,j}(r,\vartheta ) = \frac{{2\pi ab}}{{\lambda f}}\left( {\frac{r}{b}} \right)^{1 - n} \cdot \frac{{\cos \left[ {\left( {\vartheta - \left( {j - 1} \right)\frac{{2\pi }}{n}} \right)\left( {1 - n} \right)} \right]}}{{1 - n}} - k\frac{{r^2}}{{2f}}$$

and Ф_*n*_^(*j*)^ is a phase mask selecting the desired circular sector with its center at (*j* − 1)2π/*n* and a size of 2π/*n*:

12 $$\Phi _n^{(j)}\left( \vartheta \right) = \Theta \left( {\vartheta - \frac{{2\pi }}{n}(j - 1) + \frac{\pi }{n}} \right)\Theta \left( {\frac{{2\pi }}{n}(j - 1) + \frac{\pi }{n} - \vartheta } \right)$$

where Θ(·) is the Heaviside function (Θ(*x*) = 1 when *x* ≥ 0 and Θ(*x*) = 0 otherwise). According to Eq. (10), the total phase pattern of the OAM divider is basically the composition of *n* complementary and non-overlapping phase patterns, each defined over a circular sector spanning an angle equal to 2π/*n* and centered on (*j* − 1)2π/*n*, *j* = 1, …, *n*. Specifically, each zone is illuminated by 1/*n* of the input beam, and it is designed to wrap the impinging arc around the entire circle (1/*n*-fold circular-sector transformation). In addition, spatial frequency carriers {(*β*^(*j*)^, *ϑ*^(*j*)^)} are introduced in Eq. (10), to spatially separate the *n* output beams and locate them at defined positions (*ρ*^(*j*)^, *ϕ*^(*j*)^) in the focal plane according to:

13 $$\left\{ {\begin{array}{*{20}{c}} {\rho ^{(j)} = \frac{f}{k}\beta ^{(j)}} \\ {\phi ^{(j)} = \vartheta ^{(j)} = (j - 1)\frac{{2\pi }}{n}} \end{array}} \right.$$

where *j* = 1,…, *n* and *f* is the focal length of the optical transformation. Then, the phase corrector for the divider in Eq. (10) is given by the composition of *n* complementary and non-overlapping phase correctors, each defined over a circular sector spanning an angle equal to 2π/*n* and centered at the positions given by (*ρ*^(*j*)^, *ϕ*^(*j*)^):

14 $$\begin{array}{l}\Omega _{D - PC,n}(\rho ,\varphi ) = \\ \arg \left\{ {\mathop {\sum}\limits_{j = 1}^n {e^{i\Omega _{PC,1/n,j}\left( {\rho \prime _j,\varphi \prime _j} \right)}} \Phi _n^{(j)}\left( \varphi \right)} \right\}\end{array}$$

where (*ρ*'_j_, *φ*'_j_) are polar coordinates centered on (*ρ*^(*j*)^, *ϕ*^(*j*)^) given by Eq. (13), and:

15 $$\begin{array}{l}\Omega _{PC,1/n,j}\left( {\rho \prime _j,\varphi \prime _j} \right)\\ = \frac{{2\pi ab}}{{\lambda f}}\left( {\frac{{\rho \prime _j}}{a}} \right)^{1 - \frac{1}{n}} \cdot \frac{{\cos \left[ {\left( {\varphi \prime _j - \left( {j - 1} \right)\frac{{2\pi }}{n}} \right)\left( {1 - \frac{1}{n}} \right)} \right]}}{{1 - \frac{1}{n}}} - k\frac{{\rho ^2}}{{2f}}\end{array}$$

In Fig. 3c, f, numerical calculations are reported in the case of *n* = 2 and *n* = 3, showing the evolution of the beam in the range of the focal length. As expected, the input beam is split into *n* equal contributions that are mapped onto distinct circles, thus performing an *n*-fold division of the input OAM into a set of *n* OAM beams. In principle, the OAM divider can be generalized and properly designed to decompose the input OAM into many beams carrying different values of the OAM and whose total sum equals the OAM value of the input beam.

### Optical element design

**Fig. 4:**
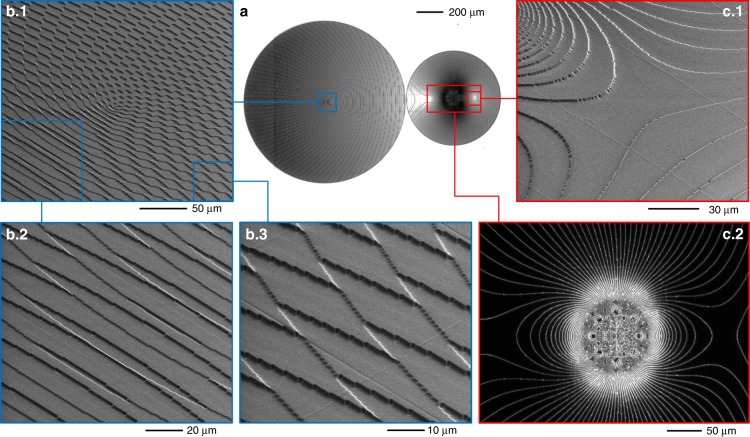
Diffractive optics for twofold multiplication. Compact twofold multiplier: inspection via optical microscope (**a**, **c**.2—top view) and SEM analysis (**b**, **c**.1—tilted views). (**b**.1) SEM inspection of the multiplier central zone and details (**b**.2, **b**.3). (**c**.2) Dark-field optical analysis of the phase-corrector central region, and further details obtained via SEM (**c**.1).

**Fig. 5:**
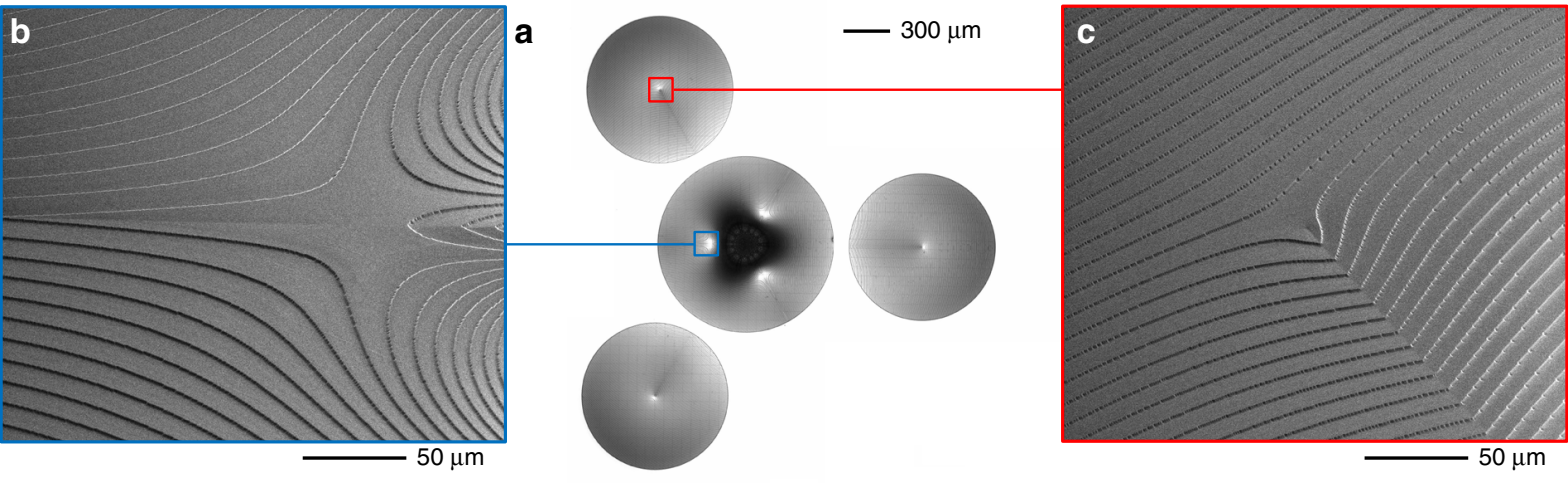
Diffractive optics for threefold division. Compact threefold divider: inspection via an optical microscope (**a**, top view) and SEM analysis (**b**, **c**—tilted views). **b** SEM inspections of the divider. **c** SEM inspection of one of the three phase correctors.

A limit of performing optical operations with transformation optics is the need for at least two optical elements, i.e., transformer and phase corrector. As widely experienced in the case of OAM sorting with a *log-pol* transformation, the presence of two confocal elements to be perfectly aligned, coaxial and coplanar, could require arduous efforts for alignment and could be detrimental to miniaturization and integration. To simplify the alignment process and improve the compactness of the optical architecture, we designed the optical configuration to incorporate the two elements onto a single optical platform. In this configuration, the substrate is illuminated twice: after crossing the first zone, while performing multiplication/division, the beam is back-reflected with a mirror, placed at half the focal length, and made to impinge on the second region providing the phase correction. By adding a tilt term to the first phase pattern, the back-reflected beam does not overlap with the input one, and the two optical elements, i.e., transformer and phase corrector, can be fabricated side-by-side on the same substrate. This solution makes the alignment operation significantly easier, as the number of degrees of freedom is remarkably reduced and the two elements are by-design aligned and parallel to each other. We fabricated compact diffractive optics performing either twofold or threefold multiplication and division. The focal length was fixed to 20 mm; therefore, the distance between the optics and the mirror was reduced to 1 cm. Adding to the first pattern a spatial frequency *β* of ~0.5 μm^−1^, the centers of the two phase elements were found to be ~1 mm from each other. For the multiplier, this result is achieved by adding a phase term *βx* to the phase in Eq. (8) and centering the phase corrector at a distance *βf/k* from the multiplier center. As far as the divider is concerned, as shown in Eq. (10), spatial frequency carriers are already included to separate the different OAM beams in the output; therefore, it is sufficient to choose the moduli *β*^(*j*)^ to avoid overlap of the phase-correcting zones with the divider pattern. The radius of the first zone was set at approximately 600 μm, whereas the radii of the phase correctors were chosen to avoid overlapping the first pattern (see Fig. 4 and Fig. 5). In more detail, for both multipliers, we set the parameters *a* and *b* to 300 μm and 250 μm, respectively, whereas for the dividers we chose the values *a* = 250 μm and *b* = 400 μm.

### Electron-beam lithography

The designed optical elements were fabricated as surface-relief phase-only diffractive optics using high-resolution electron-beam lithography (EBL) over a resist layer. By locally controlling the released electronic dose, a different dissolution rate is induced point-by-point on the exposed polymer with a resolution of few nanometers, giving rise to a spatially variant resist thickness after the development process^38^. A dose-depth correlation curve (contrast curve) is required to calibrate the correct electron dose to assign to obtain the desired thickness. For a phase pattern Ω(*x*, *y*), the depth *d*(*x*, *y*) of the exposed zone for normal incidence in air is given by:

16 $$d\left( {x,y} \right) = \frac{\lambda }{{n_R\left( \lambda \right) - 1}} \cdot \frac{{2\pi - \Omega \left( {x,y} \right)}}{{2\pi }}$$

where *λ* is the incident wavelength and *n*_*R*_(*λ*) is the corresponding refractive index of the resist. In this work, all diffractive optics were fabricated by patterning a layer of negative resist (AR-N 7720.30, Allresist) spin-coated onto a 1.1 mm-thick ITO-coated soda lime float glass substrate. At the experimental wavelength of the laser (*λ* = 632.8 nm), the refractive index of the resist polymer was assessed to be *n*_R_ = 1.679. From Eq. (16), the maximal depth of the surface-relief pattern was found to be 928.5 nm, with a thickness resolution of Δ*d* *=* 3.64 nm. The quality of the fabricated phase-only optical elements was assessed with scanning electron microscopy (SEM) and optical microscopy. In Fig. 4, SEM inspections are reported, referring to the compact two fold multiplier. In Fig. 5, the inspections refer to the compact threefold divider.

### Optical characterization

**Fig. 6:**
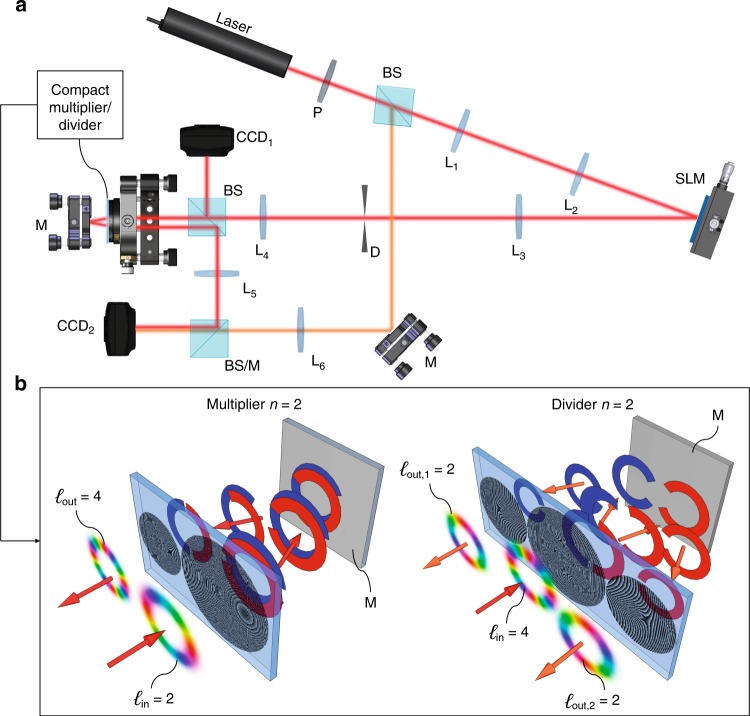
Scheme of the experimental setup. **a** Scheme of the experimental setup used for the optical characterization of the fabricated dividers and multipliers. The laser beam is linearly polarized (P) and expanded (*f*_1_ = 3.5 cm, *f*_2_ = 12.5 cm). A beam splitter (BS) is used to generate a reference Gaussian beam for the interferometric bench. The SLM first order is filtered (D) and resized (*f*_3_ = 25.0 cm, *f*_4_ = 12.5 cm) before illuminating the optical element as in **b**. A mirror (M) is used for back-reflection onto the phase-correcting pattern. A second beam splitter is used both to check the input beam and collect the output on two different cameras. The second camera is placed on the back focal plane of a fifth Fourier lens (*f*_5_ = 10.0 cm), and it is used to collect the output beam intensity and its interferogram (*f*_6_ = 20.0 cm). **b** Compact optical configurations for the twofold multiplier and the twofold divider. The two elements, i.e., tilting divider/multiplier and phase corrector, are placed on the same substrate, and a mirror (M) is used for back-reflection.

The optical response of the fabricated optical elements has been analyzed for illumination under integer OAM beams generated with an LCoS spatial light modulator (SLM) using a phase and amplitude modulation technique^39^. Once properly filtered and resized, the OAM beam illuminated the first zone of the sample, performing either *n*-fold multiplication or division, as shown in the experimental layout of the optical bench in Fig. 6a. A mirror was placed on a kinematic mount, and its position could be finely controlled with a micrometric translator to set the distance from the multiplier/divider to equal to half the focal length of the first element, i.e., 1 cm. After back reflection, the transformed beam illuminated the optics again on the phase-correcting zones, as shown in the schemes in Fig. 6b, and the far-field was collected by a second camera placed at the back focal plane of a Fourier lens. To check the OAM content of the output beams, a Mach-Zehnder interferometric bench was added to the described optical setup, as depicted in Fig. 6a.

The optical response of the fabricated optics was tested for the multiplication and division of optical beams with integer OAM values. The OAM of the output beam was analyzed from the interference pattern, with a reference arm obtained by splitting the Gaussian output of the laser. By counting the number of arms in the generated spiralgrams, it is possible to infer the OAM of the output beam and prove the expected multiplication or division of the initial OAM state. The optical characterization of the fabricated samples confirmed the expected capability to perform optical multiplication and division of the OAM of the input beam.

**Fig. 7:**
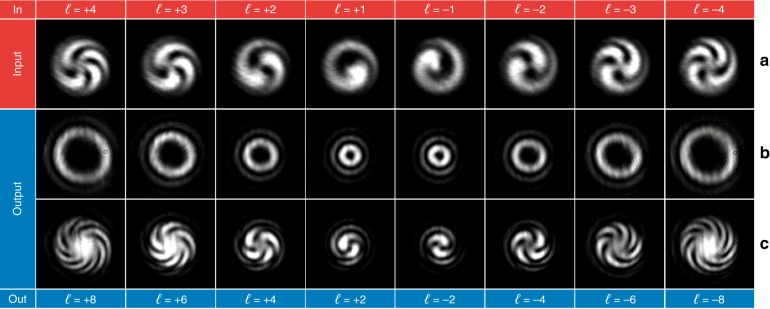
Optical characterization of the twofold multiplier for input OAM beams with *ℓ* values in the range from −4 to +4, 0 excluded. **a** Interferogram of the input OAM beams. Intensity pattern **b** and interferogram **c** of the output beam. As expected, the number of spiral arms in the output interference pattern is equal to twice the number in the input interferogram.

**Fig. 8:**
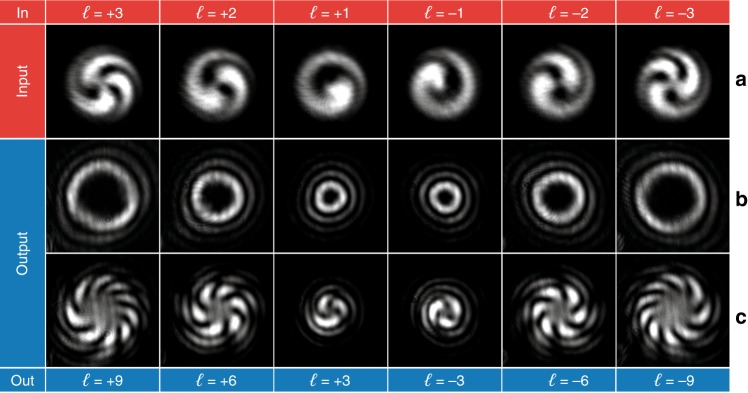
Optical characterization of the threefold multiplier for input OAM beams with *ℓ* values in the range from −3 to + 3, 0 excluded. **a** Interferogram of the input OAM beams. Intensity pattern **b** and interferogram **c** of the output beam. As expected, the number of spiral arms in the output interference pattern is equal to three times the number in the input interferogram.

In Fig. 7, the optical characterization is reported for the twofold multiplier. The optical response was characterized for input OAM beams with *ℓ* in the range from −4 to +4 (Fig. 7a), 0 excluded. As expected, the interference pattern of the output beam shows twice the number of spiral arms of the input interference pattern, confirming the duplication of the input OAM value (Fig. 7c). The same analysis was performed for the threefold multiplier for input OAM values in the range from −3 to +3, 0 excluded (Fig. 8a). As shown in Fig. 8c, the number of output arms in the interferograms is three times the number of input arms, as expected.

**Fig. 9:**
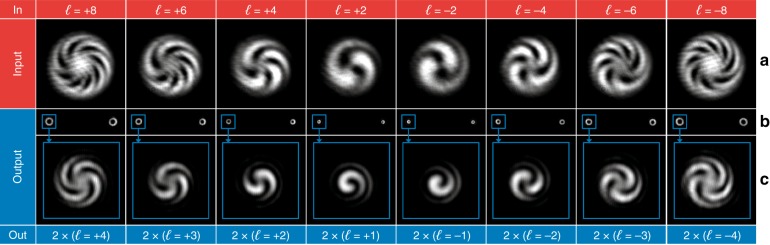
Optical characterization of the twofold divider for input OAM beams with even *ℓ* values in the range from −8 to +8, 0 excluded. **a** Interferogram of the input OAM beams. Intensity pattern **b** and interferogram **c** of the output beam. As expected, the number of spiral arms in the output interference pattern is equal to half the number in the input interferogram.

**Fig. 10:**
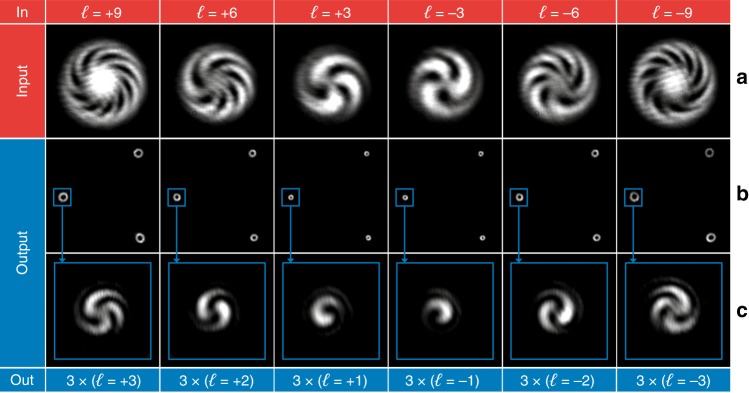
Optical characterization of the threefold divider for input OAM beams with *ℓ* values in the range from −9 to +9, with a step of 3, 0 excluded. **a** Interferogram of the input OAM beams. Intensity pattern **b** and interferogram **c** of the output beam. As expected, the number of spiral arms in the output interference pattern is equal to one-third the number in the input interferogram.

In Fig. 9, the experimental output of the twofold divider is reported for input OAM beams with even *ℓ* values in the range from −8 to +8, 0 excluded (Fig. 9a). As expected, the input beam is split into two output beams (Fig. 9b), with an OAM equal to half the input value. In Fig. 9c, the interferogram is reported for only one out of the two beams, and the interference pattern confirms the capability of the designed optics to divide the input OAM into two. The same analysis was performed for the threefold divider, as reported in Fig. 10. In this case, the output is composed of three OAM beams (Fig. 10b), each carrying one-third of the input OAM, as demonstrated by the experimental interferograms (Fig. 10c) for input *ℓ* in the range from −9 to +9, with a step of 3, 0 excluded (Fig. 10a).

## Discussion

In this paper, we presented for the first time a comprehensive work of simulation, fabrication, and optical characterization of a new type of optical device performing the division and multiplication of the orbital angular momentum of light. With respect to previous demonstrations, these optical operations were performed here in a compact and efficient configuration by means of a cascade of only two confocal optical elements. The former performs the desired optical transformation, i.e., *n*-fold multiplication or division of the input beam, whereas the latter introduces the required phase correction to correct the phase distortion accumulated during beam propagation. Both multiplication and division were implemented with a superposition of many circular-sector transformations. Specifically, multiplication by a factor *n* was realized by splitting the input OAM beam into *n* copies and mapping the whole azimuthal gradients into *n* complementary circular sectors, thus obtaining a final azimuthal gradient that is *n* times the input one. Conversely, OAM division was achieved by splitting the input azimuthal gradient into *n* complementary circular sectors and mapping them onto *n* complete circles at distinct positions.

The phase patterns of the phase-only optical elements performing twofold and threefold division/multiplication were calculated in the paraxial approximation and implemented in the form of diffractive optics fabricated with high-resolution electron-beam lithography. Furthermore, the compactness of the optical system was further improved by fabricating the two optical elements, i.e., multiplier/divider and the corresponding phase corrector, side-by-side on the same substrate, therefore reducing the number of degrees of freedom and significantly improving the alignment and miniaturization. This outcome is achievable by introducing a tilt in the first optical element and using a mirror for back-reflection onto the second pattern performing phase correction. The optical characterization with input integer OAM values confirmed the expected capability of the fabricated samples to perform either multiplication or division of the OAM content of the input beams.

As discussed in ref. ^40^ in the case of the *log-pol* optical transformer, the phase elements are designed to work perfectly for collimated beams in the input, i.e., plane wavefronts, in which all light rays illuminate the first element with normal incidence. Otherwise, it is required that the angular deviation introduced by the transformer, which is given by $\left| {\nabla \Omega } \right|/k$, dominates over any deviation from normal incidence with which the rays arrive. Irrespective of their divergence, beams carrying OAM are never plane waves, as it is the azimuthal component of the Poynting vector that originates the orbital angular momentum in the propagation direction, introducing a local skew angle around *ℓ/kr*^41^. It follows that for the multiplier/divider optics to work efficiently for an OAM beam of radius *r*, it is required that $\ell /kr \, < < \, a\left( {r/b} \right)^{ - 1/n}/f$, which provides the condition $\ell \, < < \, kab\left( {r/b} \right)^{1 - 1/n}/f$ (see Supplementary Material S3 for more details on calculations). Therefore, by properly designing the optical elements in terms of the transformation parameters (*a, b*) and focal length *f*, it is possible to increase the input OAM value, which can be either multiplied or divided with minimal distortion and maximal conversion efficiency.

So far, the possibility of increasing and decreasing the OAM content of a beam in a multiplicative manner was still missing; therefore, the framework offered by these optical elements can offer interesting and promising applications in a wide range of fields, both in the classical and single-photon regimes. OAM multiplication could find applications in the generation of high-order OAM states, for structured-light studies, or for incremental particle tweezing and trapping/manipulation. By using many low-order multipliers in cascades, it could be possible to easily increase the OAM by one order of magnitude or more. In this case, the design of the optics should be properly optimized to extend the OAM bandwidth and allow operation on high OAM values without dramatic distortions. By increasing the input OAM using a twofold or threefold multiplier before using a standard *log-pol* sorter, it could be possible to significantly enhance the cross-talk by improving the channel separation without the need for a fan-out optical operation. As a matter of fact, OAM multiplication and division are also expected to work for OAM-beam superposition (see Supplementary Material S4). Multipliers and dividers could represent the key elements needed to implement OAM algebra in optical boards performing computations with the OAM of light. Moreover, they can allow the compact and efficient implementation of the operations required in next-generation optical platforms performing switching and routing between OAM channels in mode-division multiplexing architectures. In this paper, OAM division was limited to an equalized set of output modes; however, it could be generalized for the generation of any complementary set of either integer or fractional OAM states.

The theory underlying the division and multiplication of azimuthal phase gradients, peculiar of modes endowed with orbital angular momentum, can be extended beyond the specific optical range of the presented study. For instance, the exploitation of mode-division multiplexing for high-capacity transmissions in free space has acquired increasing attention in the microwave regime, and the implementation of diffractive optics for OAM control and manipulation has recently been shown^42^. Moreover, the interest in physical states carrying OAM has recently encompassed other branches of physics by applying the results of optics to quantum mechanical wave functions describing massive particles (matter waves)^43^. In particular, the possibility to control and analyze the OAM of free-electron beams has recently been investigated with diffractive^44,45^ or electrostatic phase^46^ elements. Therefore, we envisage that similar devices performing multiplication/division could be developed for studies concerning the multiplicative transformation of the OAM carried by twisted matter waves.

## Materials and methods

### Numerical simulations

A custom MATLAB code was implemented based on the convolution algorithm applied to the Fresnel diffraction integral^47^ to compute the propagation of an OAM beam impinging on the multiplier/divider phase pattern and its final phase and intensity distributions after illuminating the phase-corrector element. The phase patterns were defined over a mesh of 5121 × 5121 pixels with a resolution of 0.3125 μm. Simulations have been performed for several *n*-fold dividers and multipliers with different design parameters (*a, b*) and focal length values *f*.

### Electron-beam lithography

Diffractive optics were fabricated by patterning a layer of negative resist (AR-N 7720.30, Allresist), spin-coated on a 1.1 mm-thick ITO-coated soda lime float glass substrate (resist thickness of ~3 μm), and pre-baked for 30 min at 85 °C. The fabricated phase patterns were computed as matrices of pixels with a size of 0.52 μm and 256 phase levels. At the experimental wavelength of the laser (*λ* = 632.8 nm), the refractive index of the resist polymer was assessed to be *n*_R_ = 1.679 [as measured by spectroscopic ellipsometry (J.A. Woollam VASE, 0.3 nm spectral resolution, 0.005° angular resolution)]. From Eq. (16), the maximal depth of the surface-relief pattern was found to be 928.5 nm, with a thickness resolution Δ*d* *=* 3.64 nm. By using custom numerical codes, the phase patterns of the simulated optics were converted into a 3D multilevel structure, which was in turn transformed into a map of electronic doses. A dose correction for compensating for the proximity effects was required to both match the layout depth with the fabricated relief and obtain a good shape definition in correspondence to phase discontinuities. The resist exposure was performed with a JBX-6300FS JEOL EBL machine in high-resolution mode, 12 MHz, generating at 100 KeV and 100 pA an electron beam with a diameter of 2 nm, providing a resolution down to 5 nm. After exposure, a cross-linking baking was performed at 100 °C for 60 min, followed by a post-baking process at 70 °C for 2 hours to improve the surface roughness. Finally, samples were developed for 510 s in AR 300-47 developer (Allresist). After development, the optical elements were gently rinsed in deionized water and blow-dried under nitrogen flux.

### Optical characterization

Integer OAM beams were generated employing an LCoS SLM (PLUTO-NIR-010-A, Holoeye) using a phase and amplitude modulation technique^39^. The Gaussian beam (*λ* = 632.8 nm, beam waist *w*_*0*_ = 240 μm, and power of 0.8 mW) emitted by a HeNe laser (HNR008R, Thorlabs) was linearly polarized and expanded with a telescope (*f*_1_ = 3.5 cm, *f*_2_ = 12.5 cm) before illuminating the SLM display. A second telescope (*f*_3_ = 25.0 cm, *f*_4_ = 12.5 cm) was used to isolate and image the first order encoded mode onto the optical element under test, mounted on a six-axis kinematic mount (K6XS, Thorlabs). A 50:50 beam splitter was used to split the beam and check the input beam profile with the first camera (DCC1545M, Thorlabs). The beam illuminated the first zone of the sample, performing either *n*-fold multiplication or division. A mirror was placed on a kinematic mount (KM100, Thorlabs), and its position could be finely controlled with a micrometric translator (TADC-651, Optosigma). The distance from the multiplier/divider was equal to half the focal length of the first element, i.e., 1 cm. After back-reflection, the transformed beam illuminated the optics again on the phase-correcting zones, and the Fourier-transformed beam was collected by a second CMOS camera (DCC1545M, Thorlabs), placed at the back focal plane of a lens with *f*  = 10.0 cm. To check the OAM content of the output beams, a Mach-Zehnder interferometric bench was added to the described optical setup, as depicted in Fig. 6a.

## Supplementary information

Supplementary information file
