# Supplementary material for: Multiplication and division of the orbital angular momentum of light with diffractive transformation optics
Source: Light Sci Appl. 2019 Dec 5;8:113. doi: 10.1038/s41377-019-0222-2 (PMC6892886; doi:10.1038/s41377-019-0222-2)
Supplement: Supplementary file 1 — Supplementary information file [file 41377_2019_222_MOESM1_ESM.pdf]

SUPPLEMENTARY MATERIAL

Multiplication and division of the orbital angular momentum of light with diffractive transformation optics

Gianluca Ruffato[1], Michele Massari[2,3], and Filippo Romanato[1,2,3]

[1]Department of Physics and Astronomy 'G. Galilei', University of Padova, via Marzolo 8, 35131 Padova, Italy

[2]LaNN, Laboratory for Nanofabrication of Nanodevices, EcamRicert, Corso Stati Uniti 4, 35127 Padova, Italy.

[3]CNR-INFM TASC IOM National Laboratory, S.S. 14 Km 163.5, 34012 Basovizza, Trieste, Italy

Authors e-mails:

Gianluca Ruffato: gianluca.ruffato@unipd.it

Michele Massari: massari@iom.cnr.it

Filippo Romanato: filippo.romanato@unipd.it

Correspondence: Gianluca Ruffato, via Marzolo 8, Department of Physics and Astronomy 'G. Galilei', University of Padova, 35125 Padova (Italy), tel. +390498275933, fax. +390498277102

S1. CIRCULAR-SECTOR TRANSFORMATION IN THE STATIONARY PHASE APPROXIMATION

For the benefit of the reader, we provide here a more detailed calculation of the phase functions of multiplier/divider optics in the stationary phase approximation. The key-element of OAM multiplication and division is represented by an optical transformation performing a conformal mapping of the whole circle onto a circular sector, the so-called circular-sector transformation. By indicating with (r, ϑ) the polar coordinates on the input plane, and with (ρ, φ) the polar reference frame on the second plane, the transformation operates a rescaling of the azimuthal coordinate:

$$\varphi = \frac{\vartheta}{n} \tag{1}$$

The transformation of the radial coordinates can be obtained after imposing the condition of conformity to the optical transformation [1], which is equivalent to $\nabla \times \vec{\rho} = 0$, where $\vec{\rho} = \rho(\cos\varphi, \sin\varphi)$. This condition leads to the following system of equations:

$$\begin{cases} \dfrac{\partial \rho}{\partial x} = -\rho \dfrac{\partial \varphi}{\partial y} \\ \dfrac{\partial \rho}{\partial y} = \rho \dfrac{\partial \varphi}{\partial x} \end{cases} \tag{2}$$

After substituting the relation in eq. (1), and using the definition $\vartheta = \arctan(y/x)$, we obtain:

$$\begin{cases} \dfrac{\partial \rho}{\partial x} = -\dfrac{\rho}{n} \dfrac{x}{x^2+y^2} \\ \dfrac{\partial \rho}{\partial y} = -\dfrac{\rho}{n} \dfrac{y}{x^2+y^2} \end{cases} \tag{3}$$

which provides the following solution:

$$\rho = a \left(\frac{r}{b} \right)^{-\frac{1}{n}} \tag{4}$$

a and b being arbitrary scaling parameters, $r = \sqrt{x^2+y^2}$. By applying the previous transformation, an azimuthal phase gradient defined over the whole range 2π is mapped conformally onto a circular sector with amplitude $2\pi/n$. In order to calculate the phase pattern of an optical element performing this transformation in the paraxial regime, we apply the stationary phase approximation [2] to the Fresnel-Kirchhoff integral. The field $U(u,v)$ after a propagation length f for an input plane-wave illuminating a phase-only optical element with phase function $\Omega_{S,n}$, located at z=0, is given by:

$$U(u,v) = \frac{e^{ik\frac{u^2+v^2}{2f}}}{i\lambda f} \iint e^{i\Omega_{S,n}(x,y)} e^{ik\frac{x^2+y^2}{2f}} e^{-ik\frac{ux+vy}{f}} dxdy \tag{5}$$

According to the stationary phase approximation [2], a two-dimensional integral with the form:

$$U = c \int_{-\infty}^{+\infty} \int_{-\infty}^{+\infty} f(x,y) e^{i\Phi(x,y)} dxdy \tag{6}$$

can be approximated with its contributions around the saddle points of the phase function $\Phi(x,y)$, as it follows:

$$U = c \sum_{\{(x^*,y^*)\}} f(x^*,y^*) e^{i\Phi(x^*,y^*)} \frac{2\pi i \sigma}{\sqrt{|AB - C^2|}} \tag{7}$$

where $A = \partial^2\Phi / \partial x^2$, $B = \partial^2\Phi / \partial y^2$, $C = \partial^2\Phi / \partial x \partial y$, and

$$\sigma = \begin{cases} +1 & AB > C^2 \quad A > 0 \\ -1 & AB > C^2 \quad A < 0 \\ -1 & AB < C^2 \end{cases} \tag{8}$$

Therefore, the integral solution of eq. (5) reduces to find the saddle points of the phase function:

$$\Phi(x,y) = \Omega_{S,n}(x,y) + k\frac{x^2 + y^2}{2f} - k\frac{ux + vy}{f} \tag{9}$$

The condition $\nabla\Phi = 0$ leads to a system of partial derivatives of $\Omega_{S,n}$ unknown. In Cartesian coordinates, we have:

$$\begin{cases} \dfrac{\partial\Phi}{\partial x} = \dfrac{\partial\Omega_{S,n}}{\partial x} + k\dfrac{x}{f} - k\dfrac{u}{f} = 0 \\ \dfrac{\partial\Phi}{\partial y} = \dfrac{\partial\Omega_{S,n}}{\partial y} + k\dfrac{y}{f} - k\dfrac{v}{f} = 0 \end{cases} \tag{10}$$

After substituting the relations in Eqs. (1) and (4), we obtain:

$$\begin{cases} \dfrac{\partial\Omega_{S,n}}{\partial x} = k\dfrac{a}{f}\left(\dfrac{r}{b}\right)^{-\frac{1}{n}} \cos\left(\dfrac{\vartheta}{n}\right) - k\dfrac{x}{f} \\ \dfrac{\partial\Omega_{S,n}}{\partial y} = k\dfrac{a}{f}\left(\dfrac{r}{b}\right)^{-\frac{1}{n}} \sin\left(\dfrac{\vartheta}{n}\right) - k\dfrac{y}{f} \end{cases} \tag{11}$$

The integration can be done easily, remembering the definition:

$$\nabla\Omega_{S,n} \cdot \vec{r} = \frac{\partial\Omega_{S,n}}{\partial r} \tag{12}$$

being $\vec{r} = (\cos\vartheta, \sin\vartheta)$. We obtain:

$$\frac{\partial\Omega_{S,n}}{\partial r} = k\frac{a}{f}\left(\frac{r}{b}\right)^{-\frac{1}{n}}\cos\left(\frac{\vartheta}{n}\right)\cos(\vartheta) - k\frac{r}{f}\cos^2(\vartheta) + k\frac{a}{f}\left(\frac{r}{b}\right)^{-\frac{1}{n}}\sin\left(\frac{\vartheta}{n}\right)\sin(\vartheta) - k\frac{r}{f}\sin^2(\vartheta)$$
$$= k\frac{a}{f}\left(\frac{r}{b}\right)^{-\frac{1}{n}}\cos\left(\vartheta - \frac{\vartheta}{n}\right) - k\frac{r}{f} \tag{13}$$

After a straightforward integration we get the result:

$$\Omega_{S,n}(r,\vartheta) = k\frac{ab}{f}\left(\frac{r}{b}\right)^{1-\frac{1}{n}}\frac{\cos\left(\vartheta - \frac{\vartheta}{n}\right)}{1-\frac{1}{n}} - k\frac{r^2}{2f} \tag{14}$$

The second optical element, i.e. the phase-corrector, is required to compensate for the phase term in eq. (7):

$$\Omega_{PC,n}(\rho,\varphi) = -\Phi\left(r^*,\vartheta^*\right) - k\frac{\rho^2}{2f} \tag{15}$$

That is:

$$\Omega_{PC,n}(\rho,\varphi) = -k\frac{ab}{f}\left(\frac{\rho}{a}\right)^{1-n}\frac{\cos(n\varphi-\varphi)}{1-\frac{1}{n}} - k\rho\frac{b}{f}\left(\frac{\rho}{a}\right)^{-n}\cos(n\varphi-\varphi) - k\frac{\rho^2}{2f} =$$
$$= k\frac{ab}{f}\left(\frac{\rho}{a}\right)^{1-n}\frac{\cos\left((1-n)\varphi\right)}{1-n} - k\frac{\rho^2}{2f} \tag{16}$$

which is basically the same expression as in eq. (14) after the substitutions $b \to a$, $n \to 1/n$, $(r,\vartheta) \to (\rho,\varphi)$. As a matter of fact, eq. (16) can be obtained considering also the inverse path travelled by light under time reversal. In the reverse configuration, the phase-corrector works as a circular-sector transformation with a factor $1/n$, operating the optical transformation:

$$\begin{cases} \vartheta = n\varphi \\ r = b\left(\frac{\rho}{a}\right)^{-n} \end{cases} \tag{17}$$

Substituting the previous relations into eq. (11) and integrating, the expression of eq. (16) can be easily obtained.

The amplitude term in eq. (7) can be calculated from the second derivatives of eq. (9). After inserting the expression in eq. (14) and performing calculations, we find out the following amplitude modulation:

$$\frac{2\pi}{\sqrt{\left|AB - C^2\right|}} = \frac{\lambda fbn}{a}\left(\frac{\rho}{a}\right)^{-n-1} \tag{18}$$

For the sake of completeness and in order to introduce the multiplier phase function (section S2), we consider the case in which an azimuthal shift is introduced in eq. (1). The corresponding conformal transformation is given by:

$$\begin{cases} \varphi = \dfrac{\vartheta}{n} + \phi_0 \\ \rho = a\left(\dfrac{r}{b}\right)^{-\frac{1}{n}} \end{cases} \tag{19}$$

which is realized by the phase element with phase function:

$$\Omega_{S,n,\varphi_0}(r,\vartheta) = k\frac{ab}{f}\left(\frac{r}{b}\right)^{1-\frac{1}{n}} \frac{\cos\left(\vartheta - \dfrac{\vartheta}{n} - \phi_0\right)}{1-\dfrac{1}{n}} - k\frac{r^2}{2f} \tag{20}$$

Conversely, the corresponding phase-corrector performs the inverse transformation:

$$\begin{cases} \vartheta = n\varphi - n\phi_0 \\ r = b\left(\dfrac{\rho}{a}\right)^{-n} \end{cases} \tag{21}$$

which is achieved by the phase-element, i.e. the phase-corrector of eq. (20), given by:

$$\Omega_{PC,n,\varphi_0}(\rho,\varphi) = k\frac{ab}{f}\left(\frac{\rho}{a}\right)^{1-n} \frac{\cos\left((1-n)\varphi + n\phi_0\right)}{1-n} - k\frac{\rho^2}{2f} \tag{22}$$

S2. MULTIPLIER PHASE PATTERN CALCULATION

The phase pattern $\Omega_{M,n}$ of the optical element performing multiplication by a factor n is described as the superposition of n circular-sector transformations $\{\Omega_{S,n}{}^{(j)}\}$, $j=1,\ldots n$, mapping the input beam over the corresponding circular sectors with amplitude $2\pi/n$ and centered in $\{(j\text{-}1)2\pi/n\}$:

$$\Omega_{S,n}{}^{(j)}(r,\vartheta)=\frac{2\pi ab}{\lambda f}\left(\frac{r}{b}\right)^{1-\frac{1}{n}}\cdot\frac{\cos\left[\vartheta\left(1-\frac{1}{n}\right)-(j-1)\frac{2\pi}{n}\right]}{1-\frac{1}{n}}-k\frac{r^2}{2f} \tag{23}$$

where we used eq. (20). Then the multiplier phase is given by:

$$\Omega_{M,n}(r,\vartheta)=\arg\left\{\sum_{j=1}^{n}e^{i\Omega_{S,n}{}^{(j)}}\right\} \tag{24}$$

Each term $\Omega_{S,n}{}^{(j)}$ performs the circular-sector transformation with a shift term $\phi_j=(j\text{-}1)2\pi/n$:

$$\begin{cases}\varphi=\dfrac{\vartheta}{n}+\dfrac{2\pi}{n}(j-1)\\[2ex] \rho=a\left(\dfrac{r}{b}\right)^{-\frac{1}{n}}\end{cases} \tag{25}$$

Therefore, the corresponding phase-corrector is given by eq. (22), with the position $\phi_0=(j-1)2\pi/n$:

$$\Omega_{PC,n}{}^{(j)}(\rho,\varphi)=k\frac{ab}{f}\left(\frac{\rho}{a}\right)^{1-n}\frac{\cos\left((1-n)\varphi+2\pi(j-1)\right)}{1-n}-k\frac{\rho^2}{2f}=\Omega_{PC,n}(\rho,\varphi) \tag{26}$$

Due to the 2π-periodicity of the cosine, the phase-corrector is therefore invariant under azimuthal shift of integer multiples of $2\pi/n$. As a consequence, each circular-sector transformation in eq. (23) has the same phase-corrector pattern, as shown in Fig. S1 for the three-fold multiplier, and it is equal to the phase-corrector corresponding to the circular-sector transformation by a factor n.

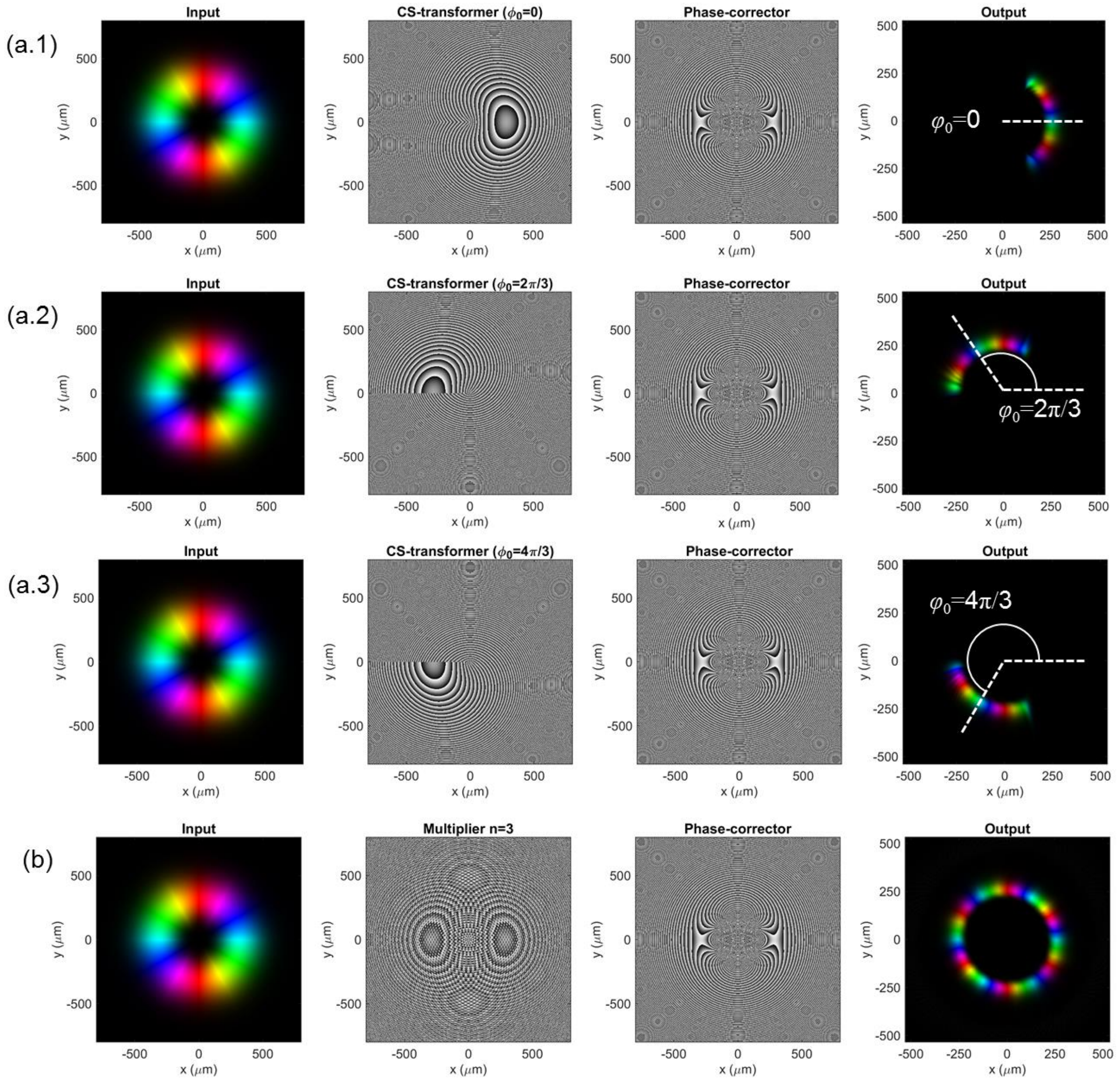

Figure S1. Construction of the three-fold multiplier (b). The phase-pattern of the first element is the combination, according to eq. (24), of three circular-sector transformations mapping the input azimuthal phase onto three non-overlapping circular sectors with amplitude $2\pi/3$, shifted of 0 (a.1), $2\pi/3$ (a.2), and $4\pi/3$ (a.3). Due to the periodicity of the phase-corrector phase function, expressed in eq. (26), the phase-corrector phase pattern is invariant under those shifts.

S3. DISTORTION OF CIRCULAR-SECTOR TRANSFORMATION FOR INPUT TWISTED LIGHT

In previous calculations in the stationary phase approximation, we assumed that the input field was impinging on the phase element with a uniform phase. This is not the case of OAM beams, in which twisted wave-fronts illuminate the optics with an azimuthal phase term $\Omega_\ell(r,\vartheta)=\exp(i\ell\vartheta)$. As already demonstrated in the case of *log-pol* optical transformation [3], the screwed wavefront introduces a distortion in the output field distribution, which is negligible as far as the input OAM is far below a threshold value. In the following, we provide an analytical model in order to show this behaviour in the paraxial regime. In the paraxial ray approximation, a ray passing through a phase mask Ω at the position (x_0, y_0), placed in the plane z=0, is deflected at an angle $1/k\cdot\nabla\Omega\big|_{x_0,y_0}$. Therefore, the intersection with a plane placed in z has the following coordinates [4]:

$$\begin{cases} x(z)=x_0+\dfrac{z}{k}\dfrac{\partial\Omega}{\partial x}\bigg|_{x_0,y_0} \\ y(z)=y_0+\dfrac{z}{k}\dfrac{\partial\Omega}{\partial y}\bigg|_{x_0,y_0} \end{cases} \tag{27}$$

If an OAM beam illuminates the optical element with a phase function $\Omega_{S,n}$ given by eq. (14), we have to include in eq. (27) the contribution of the initial phase gradient, that is $\Omega=\Omega_{S,n}+\Omega_\ell$. Due to the azimuthal phase of the OAM beam, the impinging rays acquire an input skew angle. Then, on a plane perpendicular to the propagation direction at the position z, the different rays emerging from a radial position r of the phase mask in eq. (14), intersect at the following positions, describing the parametric curve:

$$\begin{cases} x(z)=r\cos\vartheta\left(1-\dfrac{z}{f}\right)+\dfrac{az}{f}\left(\dfrac{r}{b}\right)^{-\frac{1}{n}}\cos\left(\dfrac{\vartheta}{n}\right)-\dfrac{z\ell}{kr}\sin\vartheta \\ y(z)=r\sin\vartheta\left(1-\dfrac{z}{f}\right)+\dfrac{az}{f}\left(\dfrac{r}{b}\right)^{-\frac{1}{n}}\sin\left(\dfrac{\vartheta}{n}\right)+\dfrac{z\ell}{kr}\cos\vartheta \end{cases} \tag{28}$$

where we used the definition $(x_0,y_0)=(r\cos\vartheta,r\sin\vartheta)$. At the focal plane z=f, we obtain:

$$\begin{cases} x(f)=a\left(\dfrac{r}{b}\right)^{-\frac{1}{n}}\cos\left(\dfrac{\vartheta}{n}\right)-\dfrac{f\ell}{kr}\sin\vartheta \\ y(f)=a\left(\dfrac{r}{b}\right)^{-\frac{1}{n}}\sin\left(\dfrac{\vartheta}{n}\right)+\dfrac{f\ell}{kr}\cos\vartheta \end{cases} \tag{29}$$

Considering the radial distance $R=\sqrt{x^2+y^2}$ as a function of the angle φ on the focal plane, and recalling the relation $\varphi=\vartheta/n$, we obtain:

$$R(\varphi)=\sqrt{a^2\left(\frac{r}{b}\right)^{-\frac{2}{n}}+\left(\frac{f\ell}{kr}\right)^2-2a\left(\frac{f\ell}{kr}\right)\left(\frac{r}{b}\right)^{-\frac{1}{n}}\sin\left[(n-1)\varphi\right]} \tag{30}$$

Therefore, a periodic oscillation of the ring radius is introduced, with a period equal to $2\pi/(n-1)$, i.e. decreasing with the multiplication factor n. The distortion introduced by the input screwed wavefronts is negligible, provided the following condition is satisfied $f\ell/kr << a(r/b)^{-1/n}$, which suggests a condition on the input ℓ:

$$\ell << \frac{kab}{f}\left(\frac{r}{b}\right)^{1-\frac{1}{n}} \tag{31}$$

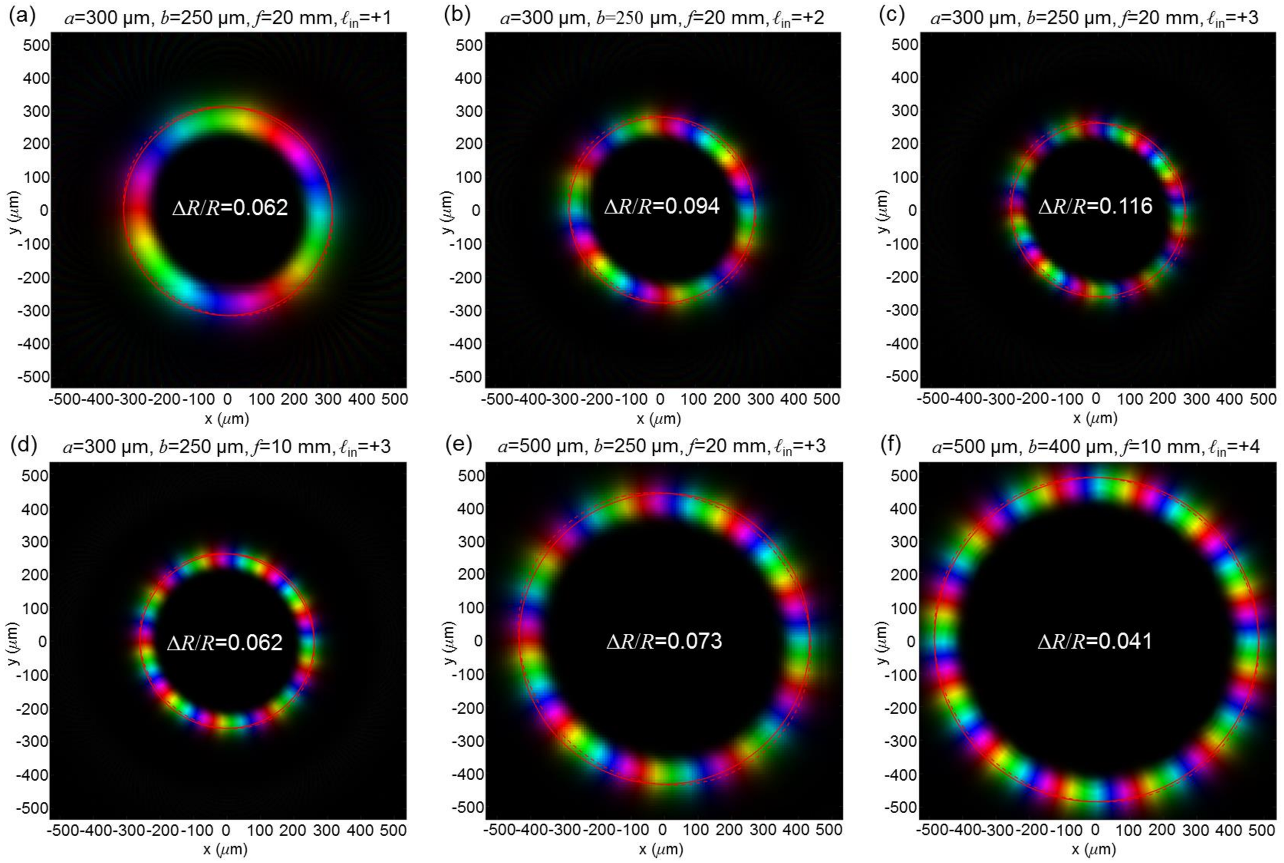

Figure S2. Three-fold multiplier. Output patterns for different input OAM values ℓ_{in} and design parameters a,b,f. Brightness and colours refer to intensity and phase, respectively. In each case, the distortion parameter $\Delta R/R$ is reported, which quantifies the deviation of the output intensity distribution (dashed red line) from the ideal one (solid red line), due to the input twisted wavefront. As expected, the distortion increases with ℓ_{in} (a-c), and it can be reduced by acting properly on the design parameters (d-f).

In Figure S2, a simulation of the output intensity distribution is performed for a three-fold multiplier (n=3) with different design parameters and input OAM values. Substituting n=3 in eq. (30), we expect that the ring radius oscillates with a period of π, exhibiting minimum values at $\varphi=\pi/4$ and $5\pi/4$, maximum values at $\varphi=3\pi/4$, and $7\pi/4$. We introduce the

distortion parameter $\Delta R/R$, given by the difference between the maximum and minimum radii, normalized by the radius of the non-distorted ring. As expected, for increasing input OAM value the distortion increases, as shown in Figures S2(a)-(c). As suggested by eqs. (30) and (31), the distortion can be decreased by reducing the focal length (Fig. S2(d)) or by increasing the parameter a ((Fig. S2(e)), or acting also on the parameter b (Fig. S2(f), for input OAM ℓ=+4). Alternatively, the size of the input beam can be increased.

S4. CONVERSION EFFICIENCY

The efficiency of OAM mode conversion has been estimated numerically by analysing the OAM spectrum of the generated beam $U(\rho,\varphi)$ after the phase-corrector. Since U is periodic in the azimuthal coordinate, it can be expanded as Fourier series:

$$U(\rho,\varphi)=\sum_{\ell=-\infty}^{+\infty} u_\ell(\rho)\exp(i\ell\varphi) \tag{32}$$

where

$$u_\ell(\rho)=\frac{1}{2\pi}\int_{-\pi}^{+\pi} U(\rho,\varphi)\exp(-i\ell\varphi)\,d\varphi \tag{33}$$

From Parseval's theorem, we have:

$$\frac{1}{2\pi}\int_{-\pi}^{+\pi} \left|U(\rho,\varphi)\right|^2 d\varphi=\sum_{\ell=-\infty}^{+\infty}\left|u_\ell(\rho)\right|^2 \tag{34}$$

Therefore, the total intensity can be expressed as:

$$I=\int_0^{+\infty}\int_{-\pi}^{+\pi}\left|U(\rho,\varphi)\right|^2 \rho\,d\rho\,d\varphi=\sum_{\ell=-\infty}^{+\infty}2\pi\int_0^{+\infty}\left|u_\ell(\rho)\right|^2\rho\,d\rho \tag{35}$$

And the normalized contribution of the mode with OAM equal to ℓ is given by:

$$\eta_\ell=\frac{I_\ell}{I}=\frac{\int_0^{+\infty}\left|u_\ell(\rho)\right|^2\rho\,d\rho}{\sum_{\ell=-\infty}^{+\infty}\int_0^{+\infty}\left|u_\ell(\rho)\right|^2\rho\,d\rho} \tag{36}$$

In Figure S3, we report the OAM spectrum analysis for different OAM beams illuminating a 2-fold (a-c) and a 3-fold (d-f) multiplier. As expected, for increasing input OAM value ℓ_{in} from +1 to +3, the distortion parameter $\Delta R/R$ increases from 0.062 to 0.139 and the conversion efficiency decreases from 0.97 to 0.86. The same trend is shown for the 3-fold multiplier with the same design parameters (a=300 µm, b=250 µm, f=20 mm) for input ℓ_{in}=+1 (d) and +2 (e). As suggested in the previous section, by properly changing the design parameters it is possible to reduce the distortion parameter and therefore improve

the efficiency conversion (f). However, the efficiency plots demonstrate the capability of the system to perform OAM multiplication with a high reliability.

For the sake of completeness, in Figures S4 and S5 we report the results of 2-fold multiplication (Fig. S4) and division (Fig. S5) for a superposition of two input OAM beams, demonstrating the validity of OAM multiplication and division also in the case of input OAM-beam superposition. The similarity between the numerical output spectrum $w^{(o)}(\ell)$ and the theoretical values $w^{(t)}(n\ell) = w^{(i)}(\ell)$, being $w^{(i)}$ the input, is defined as [5]:

$$S = \frac{\left[\sum_{\ell} \sqrt{w^{(o)}(\ell)\, w^{(t)}(\ell)} \right]^2}{\sum_{\ell} w^{(o)}(\ell) \sum_{\ell} w^{(t)}(\ell)} \tag{37}$$

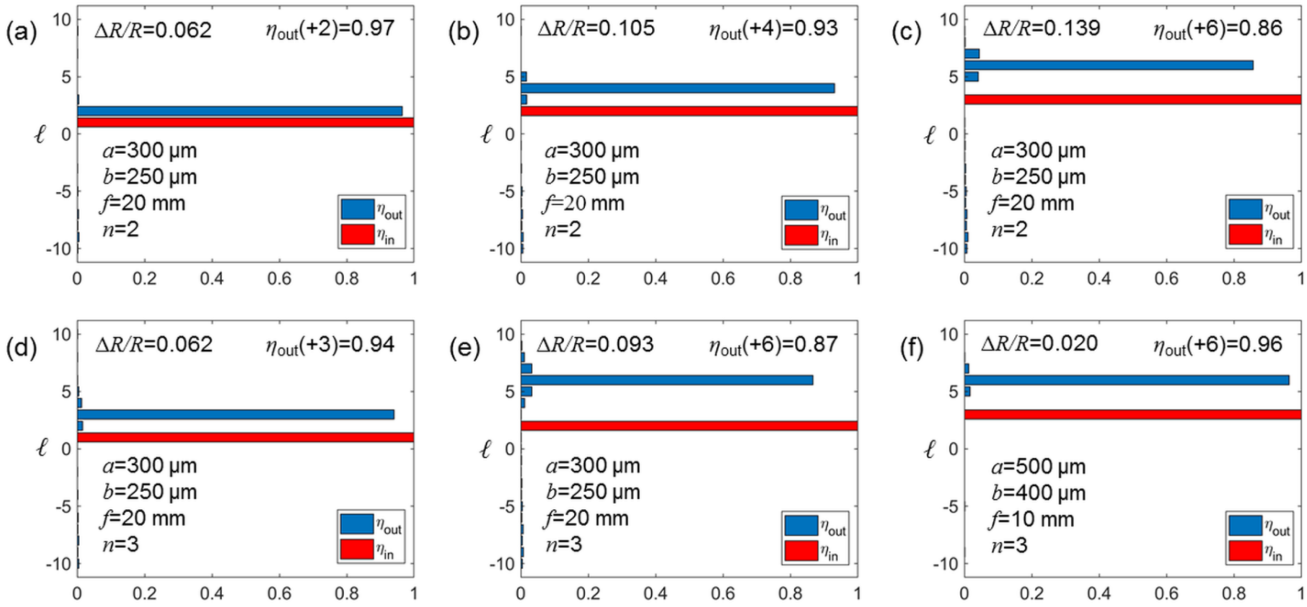

Figure S3. OAM spectrum of input (in red) and output (in blue) OAM beams for 2-fold (a-c) and 3-fold (d-f) multipliers. (a-c) 2-fold multiplier: comparison between input and output OAM spectra for increasing input OAM: ℓ_{in}=+1 (a), ℓ_{in}=+2 (b), ℓ_{in}=+3 (c). Design parameters: a=300 μm, b=250 μm, f=20 mm. (d-e) 3-fold multiplier: comparison between input and output OAM spectra for increasing input OAM: ℓ_{in}=+1 (d), ℓ_{in}=+2 (e). Design parameters: a=300 μm, b=250μm, f=20mm. (f) 3-fold multiplier with ℓ_{in}=+2. Design parameters: a=500 μm, b=400 μm, f=10 mm. For each analysis, the distortion parameter ($\Delta R/R$) and the conversion efficiency (η) are reported.

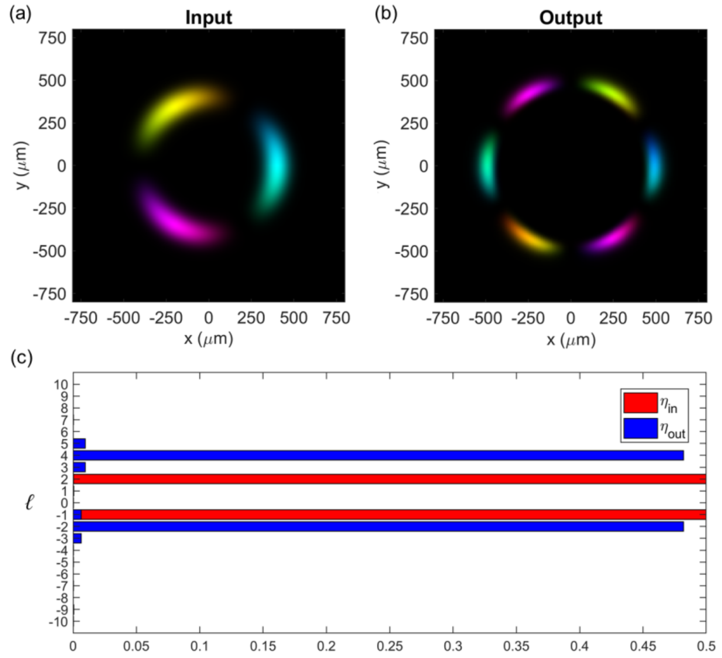

Figure S4. Two-fold multiplier: input (a) and output (b) fields for a superposition of two OAM beams with ℓ_{in}=-1 & +2. Brightness and colours refer to intensity and phase, respectively. (c) OAM spectrum of input (in red) and output (in blue) OAM-beam superposition. Design parameters: a=500 μm, b=400 μm, f=10 mm. *S*imilarity [5] between numerical and theoretical output spectra: 0.96.

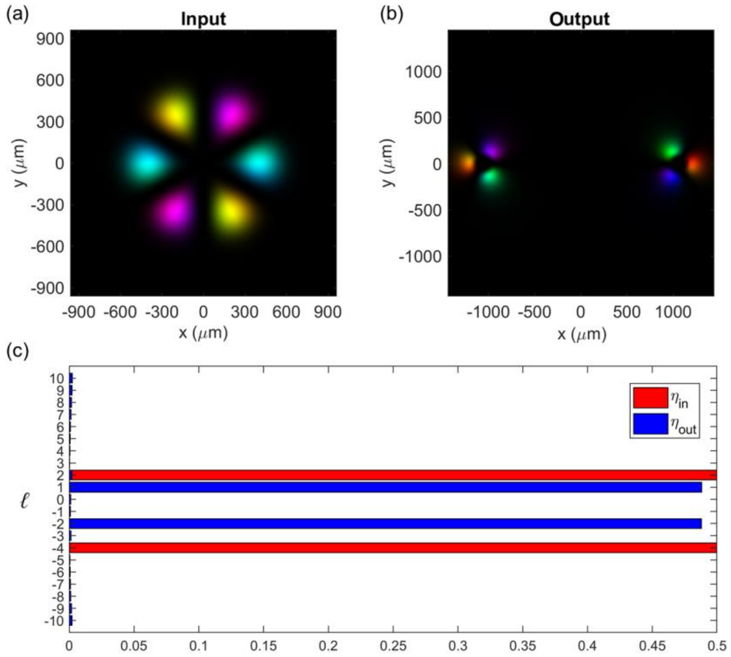

Figure S5. Two-fold divider: input (a) and output (b) fields for a superposition of two OAM beams with ℓ_{in}=-4 & +2. Brightness and colours refer to intensity and phase, respectively. (c) OAM spectrum of input (in red) and output (in blue) OAM-beam superposition. Design parameters: a=250 μm, b=500 μm, f=15 mm. *S*imilarity [5] between numerical and theoretical output spectra: 0.98.

REFERENCES:

[1] Hossack, W. J., Darling, A. M., and Dahdouh, A. Coordinate transformations with multiple computer-generated optical elements. *J. Mod. Opt.* **34**, 1235-1250 (1987).

[2] Born, M., and Wolf, E. *Principles of optics* (Pergamon Press, 1980).

[3] Lavery, M.P.J., Robertson, D.J., Sponselli, A., Courtial, J., Steinhoff, N.K., Tyler, G.A., Willner, A.E, and Padgett, M.J. Efficient measurement of an optical orbital-angular-momentum spectrum comprising more than 50 states. *New J. Physics* **15**, 013024 (2013).

[4] Greenfield, E., Segev, M., Walasik, W., and Raz, O. Accelerating light beams along convex trajectories. *Phys. Rev. Lett*. **106**, 213902 (2011).

[5] Ruffato, G., Girardi, M., Massari, M., Mafakheri, E., Sephton, B., Capaldo, P., Forbes, A., and Romanato, F. A compact diffractive sorter for high-resolution demultiplexing of orbital angular momentum beams. *Sci. Rep*. **8**, 10248 (2018).
